# Meningeal lymphatics orchestrate vertebral neutrophils via a Hex1Cer-NETs axis to promote autoimmune neuroinflammation

**DOI:** 10.1186/s12974-026-03994-x

**Published:** 2026-08-13

**Authors:** Wenxuan Sun, Hanzhang Tan, Shishi Shen, Li Xiao, Yanyu Chang, Yuge Wang, Yipeng Zhao, Jiaqi Zhang, Shilin Wu, Zihong Wang, Zhengqi Lu, Wei Cai, Wei Qiu

**Affiliations:** Department of Neurology, Mental and Neurological Disease Research Center, the Third Affiliated Hospital of Sun Yat-sen University, 600 Tianhe Road, Guangzhou, Guangdong 510630 China

**Keywords:** Multiple sclerosis, Meningeal lymphatic vessel, NETs formation, Hex1Cer

## Abstract

Meningeal enhancement is observed on cranial and spinal magnetic resonance image in a subset of patients with multiple sclerosis (MS) and is associated with a higher risk of future disease progression, however, the underlying mechanisms by which it promotes such progression remain elusive. By cross-validating clinical MRI datasets with the experimental autoimmune encephalomyelitis model (EAE), we revealed a meningeal lymphatics-innate immune axis that orchestrates a localized signaling cascade driving and promoting the pathogenesis of inflammatory demyelination. We show that the earliest phase of the disease is characterized by a rapid, selective influx of neutrophils recruited directly from the vertebral bone marrow via specialized osteo-channels. Crucially, we identify the glycosphingolipid hexosylceramide (Hex1Cer), which is derived from meningeal lymphatic vessels after drainage of myelin debris and triggers neutrophil extracellular trap (NET) formation. These meningeal NETs utilized a SELPLG-SELL axis to recruit pathogenic lymphocytes, promoting their infiltration into the spinal cord parenchyma. Both pharmacological targeting of the Hex1Cer or NETs formation pathway significantly reduced downstream T cell infiltration and clinical severity. Finally, we demonstrate that low-dose intrathecal repurposing of Sivelestat to target this meningeal niche provides superior protection compared to systemic delivery. Our findings define the meningeal lymphatic endothelial cells (mLECs) as active metabolic gatekeepers of neuroinflammation and establish the mLEC-myelin-Hex1Cer-NETs formation axis as a high-priority therapeutic target for intercepting MS.

**Supplementary Information:**

The online version contains supplementary material available at https://doi.org/10.1186/s12974-026-03994-x.

## Introduction

Multiple sclerosis (MS) is a chronic autoimmune disorder of the central nervous system (CNS), classically defined by the infiltration of pathogenic T and B lymphocytes across the blood-brain barrier (BBB), leading to focal demyelination and axonal loss [1, 2]. While adaptive immune effector mechanisms are well-characterized, the early events that breach CNS immune privilege to establish a pro-inflammatory niche remain elusive. Clinical heterogeneity in MS, spanning relapsing-remitting (RRMS) and progressive (PPMS and SPMS), is increasingly linked to meningeal pathology [3]. Notably, meningeal enhancement (ME) on post-contrast MRI [4] and the presence of paramagnetic rim lesions [5] correlate partly with cortical atrophy and accelerated disability [6–8], suggesting the meninges function as an active immunopathological interface rather than a purely structural boundary.

Recent evidence identifies CNS border tissues, particularly the meninges, as critical epicenters of early immune activity [9–11]. Anatomically, the meninges serve as a specialized conduit for immune cell trafficking from the skull and vertebral bone marrow via specialized osteo-channels [12–15], providing a direct route to the CNS that bypasses systemic circulation [16]. Parallel to these findings, the meningeal lymphatic system has emerged as a pivotal regulator of CNS surveillance. While meningeal lymphatic vessels (mLVs) are known to facilitate the trafficking [17, 18] and clearance of metabolic waste [19–21], such as amyloid-β [22], myelin debris and their byproducts are reported and found once in the meninges and peri-vascular space [23] which is regarded as part of glymphatic system. Whether mLVs act merely as passive drainage routes or as active orchestrators of the inflammatory cascade remains a central question in neuroimmunology [24].

Recent studies have demonstrated that innate immune cells originating from the cranial bone marrow infiltrate the meninges [25, 26], thereby driving the pathogenesis and progression of multiple sclerosis (MS) [25]. However, historically neutrophils which are the fastest responder to the inflammation [27], were dismissed as short-lived, nonspecific effectors in MS. However, emerging data suggest a more nuanced role. Neutrophils can undergo NETs formation, which means the release of NETs composed of decondensed chromatin and antimicrobial proteins [27], are essential for host defense and can drive autoimmune pathology [28–30]. Clinical evidence revealed a high level of neutrophils in the blood, hints at a pathogenic contribution [31]. While we previously observed NETs during the peak phase of experimental autoimmune encephalomyelitis (EAE) [31], their contribution to the subclinical initiation phase remains to be resolved.

Here, we demonstrate that the meningeal lymphatic vasculature is an active driver of early MS pathology. We provide evidence from MS patients and experimental autoimmune encephalomyelitis (EAE) models that early meningeal inflammation is characterized by a rapid, selective influx of neutrophils recruited directly partly from the vertebral bone marrow. We show that mLECs are metabolically reprogrammed by myelin debris to synthesize the Hexosylceramide (Hex1Cer). This LEC-derived lipid acts as a molecular trigger for NETs formation. Finally, we demonstrate that these meningeal NETs act as a pathogenic scaffold that recruit adaptive neuroinflammation by retaining encephalitogenic T cells via a SELPLG-SELL adhesion axis. Our findings identify the mLEC-Hex1Cer-NETs formation axis as a critical, targetable checkpoint in the transition from local innate activation to systemic autoimmune demyelination.

## Results

### Meningeal NETs is an early pathological feature of EAE and correlates with demyelination severity

**Fig. 1:**
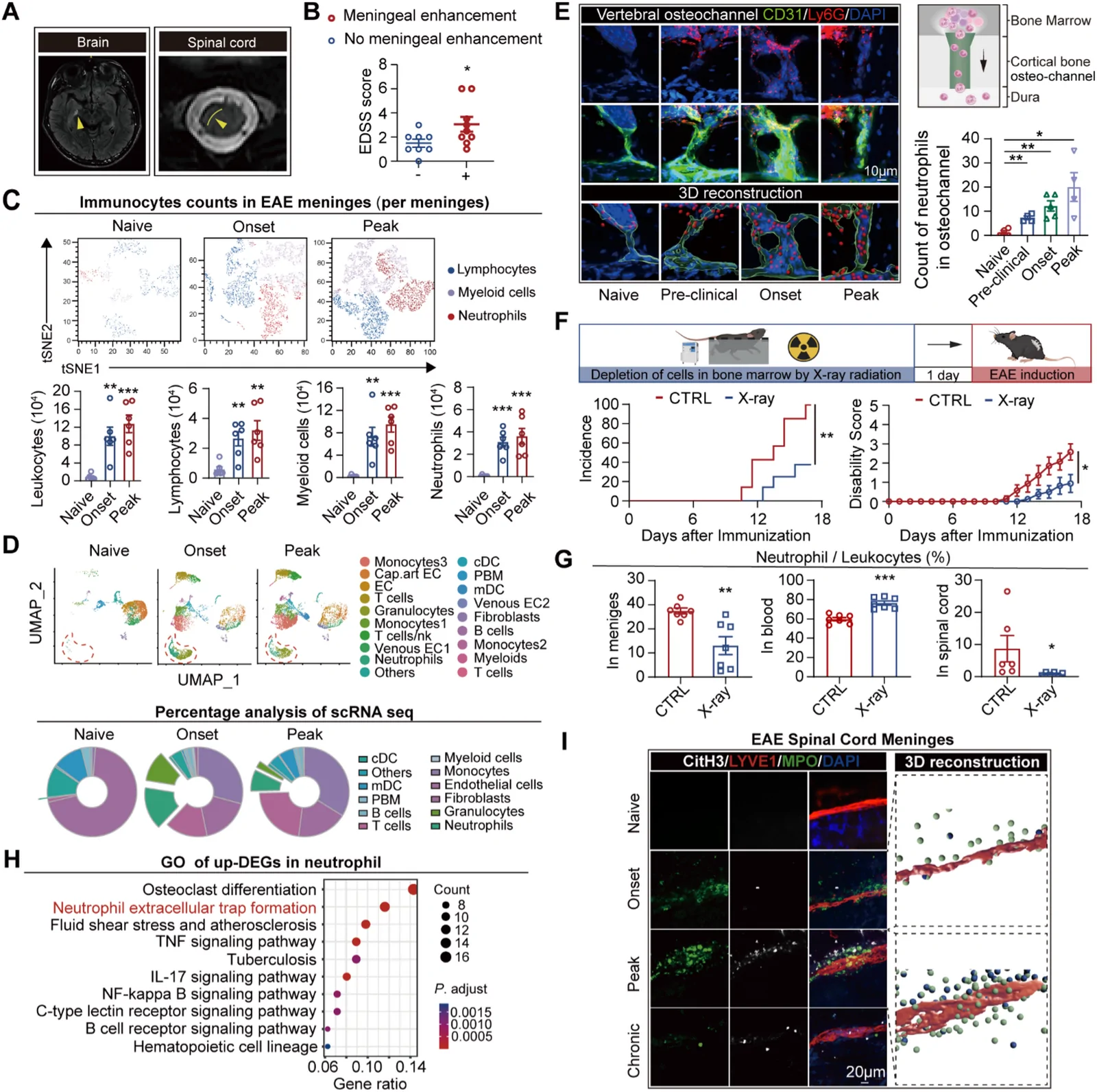
Neutrophils from vertebral bone marrow infiltrated meninges in the early stage of EAE. **A**, **B** Postcontrast MRI of MS patient demonstrating meningeal enhancement. Left panel: Brain imaging using T2 TSE dark fluid sequence. Right panel: Spinal cord imaging using T1 TSE sequence. Comparison of EDSS scores during relapse in patients with (+) or without (-) meningeal enhancement. Student’s *t*-test, N = 8, * *p* < 0.05. **C** tSNE map of flowcytometry revealed leukocytes infiltration in the meninges of different stages (Naïve, Onset and Peak) in EAE. Counts of CD45^+^ leukocytes, CD3^+^/B220^+^CD11b^-^ lymphocytes, CD11b^+^ myeloid cells and CD11b^+^Ly6G^+^ neutrophils increased obviously at the onset and peak. One-way *ANOVA*, N = 6, ** *p* < 0.01, *** *p* < 0.001. **D** UMAP and percentage analysis of single cell RNA sequence displayed neutrophils infiltrated meninges of EAE at the beginning of demyelination. **E** IF and schematic diagram showed neutrophils migrating in the CD31^+^ osteo-channel. Counts of neutrophils in the osteo-channels suggested a gradual increase as the disease progress. Scale bar = 10 μm. N = 5, One-way *ANOVA*, * *p* < 0.05. **F** Schematic diagram displayed EAE induction was 1 day after irradiation. Incidence and disability score showed decreased after irradiation. N = 6 or 7, Repeated measures two-way *ANOVA*, * *p* < 0.05, ** *p* < 0.01. **G** Flowcytometry of neutrophils in meninges, blood and spinal cord over total leukocytes. Radiation decreased influx of neutrophils in meninges and spinal cord but not in blood. N = 6 or 7, One-way *ANOVA*, * *p* < 0.05, ** *p* < 0.01, *** *p* < 0.001. **H** GO enrichment analysis suggested higher expression of “Neutrophil extracellular trap formation”. Log2Foldchange > 1, *P*.adjust < 0.05. **I** IF and 3D reconstruction showing NETs (CitH3, white) localized near meningeal lymphatic vessels (LYVE1, red) at disease onset and persisting through peak and chronic stages. Neutrophils were labeled with MPO (green). Scale bar = 20 μm

To investigate the clinical relevance of meningeal inflammation in MS, we first analyzed contrast-enhanced MRI data from a cohort of patients at the Third Affiliated Hospital of Sun Yat-sen University. Baseline characteristics of the patients are presented in Supplementary Table 1. By stratifying patients based on the presence of meningeal enhancement (ME) at relapse onset, we confirmed a more disease severity in the patients with ME, as measured by the Expanded Disability Status Scale (EDSS) (Fig. 1A-B). Having established the clinical significance of meningeal pathology, we sought to identify the early cellular drivers of this process using the MOG(35–55)-induced EAE model. We profiled the meningeal immune landscape across three stages: naive, onset, and peak (Fig. 1C-D). Consistent with the established classification of experimental autoimmune encephalomyelitis (EAE) kinetics, mice were sequentially evaluated and harvested at distinct phases: (1) Naïve Stage: Age-matched healthy female mice without immunization, analyzed at day 0. (2) Pre-clinical stage: Mice started EAE induction but without symptoms sacrificed at days 7 post immunization (dpi7). (3) Onset Stage: Defined by the initial manifestation of neurological symptoms (clinical score = 1), typically occurring around dpi12 . (4) Peak Stage: Defined by maximum neurological deficit and disease severity (clinical score ≥ 3), consistently observed between dpi 15–18. (5) Chronic stage: mice at dpi45 [32–34]. While late-stage inflammation was characterized by a broad infiltration of adaptive leukocytes such as CD3^+^ T cells, flow cytometric and unbiased single-cell RNA-sequencing (scRNA-seq) revealed a dramatic [35], disproportionate influx of CD11b^+^ Ly6G^+^ neutrophils specifically at the onset of disease (Fig. 1C-D, S1A). It was reported that skull bone marrow could produce B cells and myeloid cells in the disease condition in central nervous system [25, 36]. We wanted to further confirm the neutrophils migrating from vertebral bone marrow. Neutrophils labeled by Ly6G were found in the CD31 curved osteo-channel which connects bone marrow and meninges in the pre-clinical, onset and peak stage of EAE mice (Fig. 1E). Furthermore, we performed flow cytometric profiling and quantitative analysis of the granulocytic lineage across both femur and vertebral bone marrow in EAE mice. Prior to the manifestation of clinical symptoms, we observed a systematic shift in medullary myelopoiesis. The frequencies of granulocyte-monocyte progenitors (GMPs) were significantly expanded, whereas the mature neutrophil pool was declined (Fig.S1B). These findings indicate accelerated granulopoiesis and enhanced systemic mobilization of neutrophils from the bone marrow compartments during the pre-symptomatic phase. We further depleted bone marrow by irradiation in the vertebrae and skull bone to figure out the source and function of neutrophils. Consistent with prior reports demonstrating bone marrow depletion 5 to 7 days post-irradiation [37], our flow cytometry data showed a significant decrease in leukocytes in the vertebrae at day 5 following localized irradiation, with no observable impact on the femurs (Fig.S1C). Clinical score and incidence were alleviated after depletion, which suggested that vertebral bone marrow may participate in the inflammatory demyelination (Fig. 1F). Mice were sacrificed at the peak stage of CTRL group at dpi17. We found infiltration of neutrophils were down-regulated in the meninges while not in the blood, which claimed neutrophils from vertebrae did contribute to the meninges neutrophil pool or even to the disease course (Fig. 1G). Although we cannot rule out the possibility that irradiation also affects the pathogenic role of other bone marrow-derived immune cells, as reflected by reduced spinal cord infiltration of total leukocytes, microglia, and T cells, along with decreased meningeal T cells following radiation (Fig. S1D-G), the marked enhancement of granulopoiesis during the preclinical phase, coupled with the early infiltration of neutrophils into the meninges, prompted us to hypothesize that this vertebral bone marrow-derived neutrophil population exerts a potential role in driving disease onset and progression. To determine the functional state of these early-arriving neutrophils, we performed Gene Ontology (GO), which identified a highly significant enrichment of the “Neutrophil Extracellular Trap Formation” pathway **(**Fig. 1H). We validated this in situ using 3D reconstruction of meninges. Immunofluorescence staining for myeloperoxidase (MPO) and citrullinated Histone H3 (CitH3), a definitive hallmark of NETs, revealed extensive NETs-like structures spatially concentrated around meningeal lymphatic vessels as early as disease onset (Fig. 1I). Immunofluorescence staining demonstrated that NETs predominantly clustered adjacent to the meningeal lymphatic vessels during the early stage of the disease (Fig. S2A). Notably, NETs were largely absent from the spinal cord parenchyma at this stage (Fig. S2B), suggesting that the meninges serve as the primary and initial site for NETs formation. To further resolve this spatiotemporal discrepancy, we quantified meningeal NET levels across different disease stages using flow cytometry. Consistent with our single-cell RNA sequencing (scRNA-seq) findings, a significant elevation in meningeal NETs was detected as early as the onset phase (Fig. S2C-D). Finally, pathway enrichment analysis indicated that these meningeal neutrophils are also characterized by activated “IL-17 signaling pathways” and “B-cell receptor signaling pathways”. This molecular profile suggests that early meningeal NETs may not be a passive byproduct of inflammation, but rather a provocative event that facilitates the recruitment of adaptive immune cells and promotes subsequent inflammatory demyelination. Early-stage NETs likely bridge innate and adaptive immunity, facilitating subsequent lymphocyte recruitment. This initiating capacity aligns with prior evidence that target elimination of NETs successfully reduces central nervous system T cell infiltration at the peak of EAE [38], validating the direct influence of innate NETs on the adaptive immune landscape. These data collectively suggest that neutrophil accumulation and NETs formation are among the earliest pathological events in EAE, preceding both the peak of clinical symptoms and the massive infiltration of the CNS by adaptive immunity.

### Blockade of meningeal lymphatic function inhibits neutrophil recruitment, NETs formation, and ameliorates EAE

**Fig. 2:**
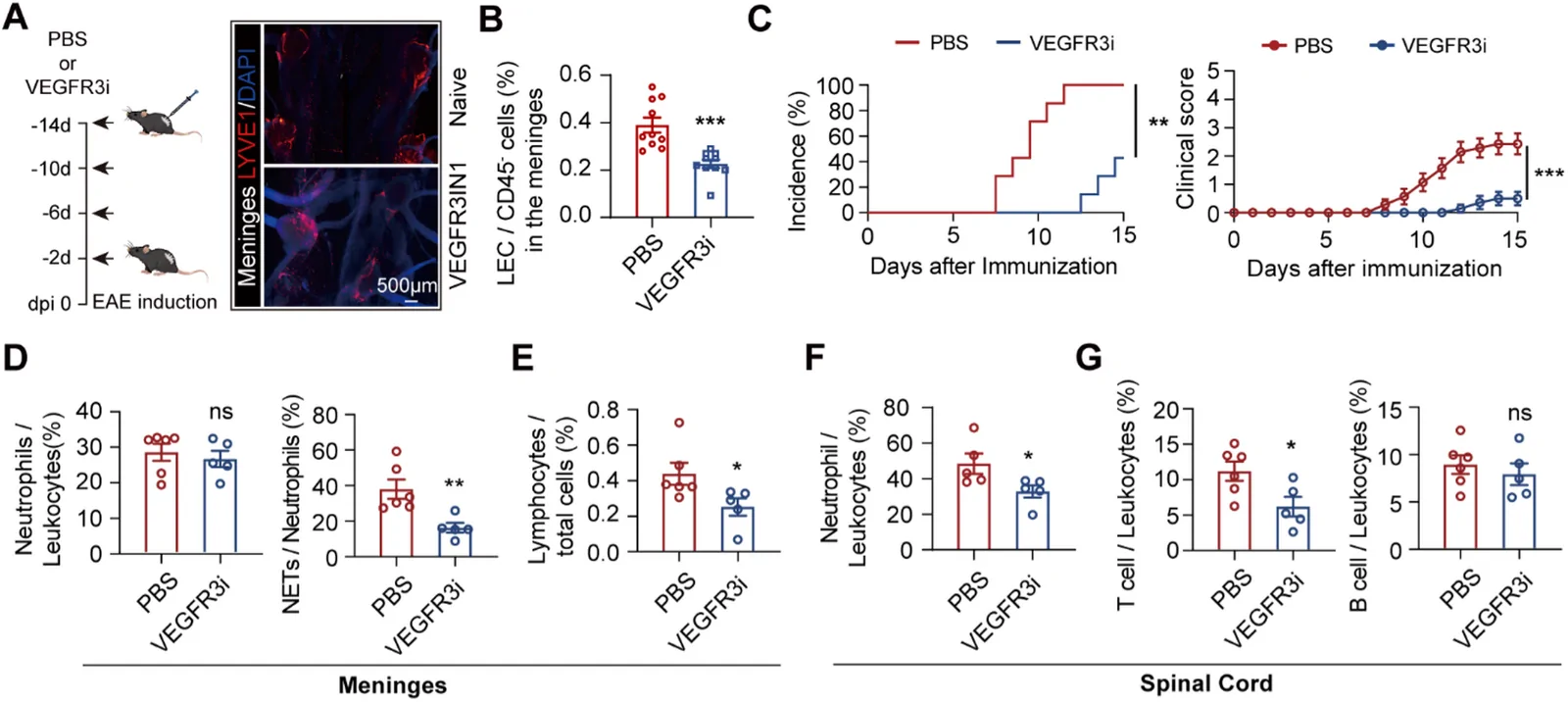
Blockade of mLVs inhibited NETs formation and ameliorates EAE. **A **Schematic of intrathecal injection schedule: VEGFR3i or PBS was administered every 4 days, starting 2 weeks before EAE induction. Intrathecal VEGFR3-IN-1 treatment reduced the distribution and altered the luminal structure of LYVE1^+^ meningeal lymphatic vessels, as shown by immunofluorescence staining (Naive VS. VEGFR3IN1). Scale bar =  500 μm. **B** Flow cytometry analysis of Live/Dead⁻CD45⁻CD31⁺LYVE1⁺PDPN⁺ lymphatic endothelial cells confirmed VEGFR3 inhibition efficiency. One-way *ANOVA*, N = 10, *** *p* < 0.001. **C** EAE incidence and clinical scores in PBS- and VEGFR3i-treated groups. VEGFR3i treatment significantly delayed disease onset and reduced severity. Repeated measures two-way *ANOVA*, N = 6, ** *p* < 0.01, *** *p* < 0.001. **D** Flow cytometry showed lower NETs formation without decreased neutrophil infiltration on the meninges in the VEGFR3i group compared with control group. One-way *ANOVA*, N = 6, ** *p* < 0.01, ns means not significant. **E**-**F** Flow cytometry revealed reduced infiltration of lymphocytes in the meninges and neutrophils in the spinal cord, along with decreased NETs formation following VEGFR3i treatment. One-way ANOVA, N = 6, * *p* < 0.05, ** *p* < 0.01. **G** In spinal cord infiltration of T lymphocytes decreased while B lymphocytes remained. One-way *ANOVA*, N = 6, * *p* < 0.05, ns means not significant

We initially investigated whether meningeal mLECs directly modulate neutrophil trafficking. Analysis of single-cell RNA sequencing data revealed a significant upregulation of *S100a8* and *S100a9* transcripts in mLECs during the onset and peak phases of EAE compared to healthy mice (Fig.S3A). To validate these transcriptomic shifts, but negatively, immunofluorescence staining denied a gradual increase in S100A9 protein expression within the mLECs as the disease advanced (Fig.S3B). Notably, during the onset phase, we identified clusters of S100A9^+^ neutrophils specifically sequestered around the mLVs, suggesting a localized recruitment or drainage axis at these perivascular sites. To determine the functional requirement of the meningeal lymphatic vasculature in this early innate immune cascade, we treated EAE mice intrathecally with a potent 0.83 mg/kg VEGFR3 inhibitor to compromise LEC integrity and signaling (Fig. 2A). Since intrathecal injection directly delivers the drug into the cerebrospinal fluid bypassing the blood-brain barrier (BBB), the systemic dose was converted to the local dose. VEGFR3 is the receptor of VEGF C and promote the proliferation and fundamental function of mLVs [39]. VEGFR3 inhibition resulted in a slight regression of the meningeal lymphatic network as confirmed by CD45⁻CD31^+^ LYVE1^+^ Podoplanin (PDPN)^+^ staining with no significant effect of CD31^+^ blood endothelial cells and CD45^+^ leukocytes (Fig. 2A-B, S3E-F). Clinically, sacrificed while CTRL started the plateau phase, mice treated with the inhibitor exhibited a markedly delayed disease onset and a profound reduction in peak clinical scores compared to controls (Fig. 2C). We then analyzed the immune cell composition to understand the mechanism of this protection. Intriguingly, while the total number of Ly6G^+^ neutrophils infiltrating the meninges at disease peak was not significantly altered by VEGFR3 inhibition (Fig. 2D), the functional phenotype of these cells was fundamentally transformed. We observed a large part absence of CitH3^+^ NETs within the meninges of treated mice (Fig. 2D). This suggests that while VEGFR3 inhibitor may not block the neutrophils recruitment, the lymphatic endothelium provides the essential second signal or physical scaffold required for the induction of NETs formation. The impact of lymphatic vessels mediated NETs formation extended beyond the meninges and neutrophil infiltration into the spinal cord parenchyma was significantly attenuated in the treated group (Fig. 2F, S3C-D). Ultimately, this early disruption of the innate immune cascade led to a profound downstream reduction in the accumulation of pathogenic CD3^+^/B220^+^CD11b^-^lymphocytes in both the meninges and the spinal cord (Fig. 2E and G). These findings demonstrate that meningeal lymphatic vessels are not merely passive conduits but are active pathological hubs that trigger NETs formation, thereby assist the transition from local innate inflammation to systemic autoimmune demyelination.

### Myelin debris drained by lymphatics activates LECs to promote NETs formation

**Fig. 3:**
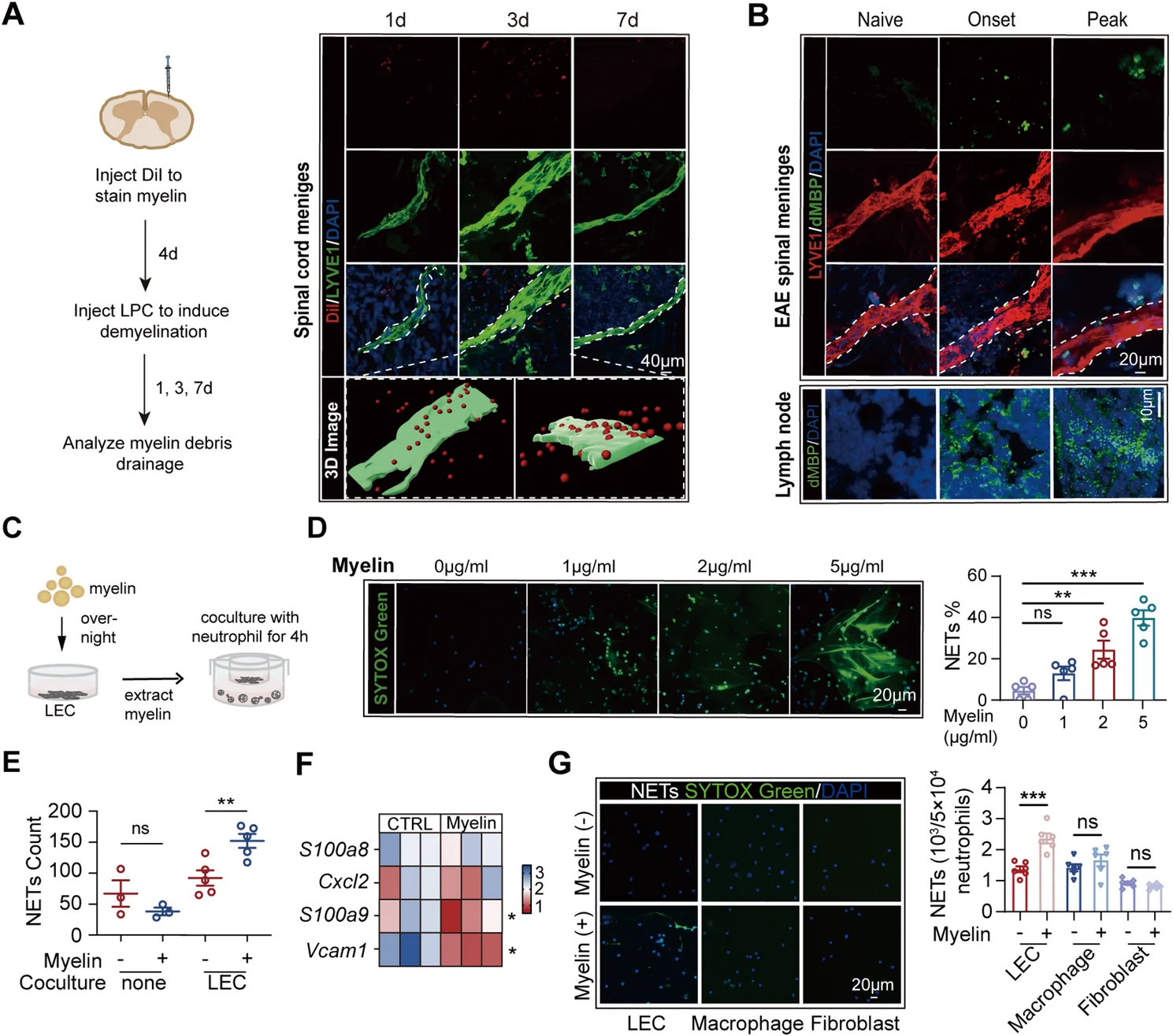
Myelin debris drained by lymphatics activates LECs to promote NETs formation. **A** Schematic of the partial demyelination model in the spinal cord. DiI (red) was injected to label myelin debris 4 days prior to lysophosphatidylcholine (LPC) injection at the same site. Spinal cord dura was collected for immunofluorescence (IF) at 1, 3, and 7 days post-induction. Lymphatic vessels labeled with LYVE1 (green). Scale bar = 40 μm. **B** Spinal cord meningeal lymphatic vessels in the EAE model at different disease stages (naïve, onset and peak), showing myelin debris (red) drainage. Lymphatic vessels labeled with LYVE1 (green). Scale bar = 20 μm. Increased myelin debris drainage in lymph nodes during EAE onset and peak stages compared to the naïve. Scale bar = 10 μm. **C** Schematic of coculture assay: LECs were pretreated with 5 µg/ml myelin debris for 24 h, washed, and cocultured with neutrophils for 4 h. **D** SYTOX Green staining indicated dose-dependent NETs formation in response to myelin debris (1–5 µg/ml). Scale bar = 20 μm. Analysis of IF confirmed increased NETs at 5 µg/ml. Two-way *ANOVA*, N = 5, ** *p* < 0.01, *** *p* < 0.001. **E** No significant NETs induction when neutrophils were directly treated with 5 µg/ml myelin debris. Two-way *ANOVA*, N = 3. **F** After treated with myelin overnight, LECs expressed higher level of *S100a9* and *Vcam1*, which was in accordance with the result of former single cell RNAseq. One-way *ANOVA*, N = 3, * *p* < 0.05. **G** Flow cytometry and immunofluorescence evaluation of NET generation. Following overnight stimulation with 5 µg/ml myelin debris and subsequent washout, mLECs, macrophages, or fibroblasts were co-cultured with neutrophils for 4 h. Elevated NET formation was exclusively observed in the myelin-pretreated mLEC group. Scale bar = 20 μm. Two-way *ANOVA*, N = 6, *** *p* < 0.001, ns means no significant

We next sought to identify the upstream signal that initiates meningeal lymphatic endothelial cells (mLECs) activation during EAE. We hypothesized that myelin debris, generated during early subclinical demyelination, is drained via the meningeal lymphatic vessels (mLVs) and acts as a localized danger signal. To test this, we injected DiI to label myelin and induced local demyelination by LPC into the spinal cord parenchyma and we observed drainage of the fluorescent debris by LYVE1^+^ LECs, positioning these cells as one of the sensors of CNS-derived pathological byproducts (Fig. 3A). This was further corroborated by staining for degraded myelin basic protein (dMBP), which revealed aggregates within the lumbar lymph nodes during the onset and peak stages of EAE (Fig. 3B). To confirm this is a conserved CNS-wide mechanism, we utilized the cuprizone-induced demyelination model. Myelin debris was similarly drained to the deep cervical lymph nodes (dCLNs) via brain mLVs (Fig.S4A-B). Notably, ligation of the dCLNs resulted in a significant accumulation of myelin debris within the mLV lumen, confirming the direct trafficking of myelin through this lymphatic route (Fig. S4A-B). To directly test whether myelin debris activates LECs to promote inflammation, we established an in vitro coculture model. Isolated myelin debris derived from rodent brain containing key myelin proteins (such as MBP, MOG, and PLP) and lipid matrices, were introduced to treat mLECs. mLECs were stimulated with low-concentration myelin debris, mimicking the low levels in mLVs rather than the high concentrations found at lesion cores. Bone marrow-derived neutrophils were then exposed to the conditioned media (CM) from these myelin-primed LECs (Fig. 3C). Remarkably, CM from myelin-stimulated LECs, but not from control LECs, robustly induced NETs formation, as quantified by SYTOX Green assays and CitH3/MPO expression (Fig. 3D, Fig. S4C). This induction followed a dose-dependent relationship with the initial myelin concentration (Fig. 3D). Crucially, a low concentraion of 5µg/ml myelin debris applied directly to neutrophils failed to induce NETs formation (Fig. 3E). Furthermore, direct myelin stimulation of LECs induced a significant upregulation of *S100a9 *and* Vcam1*, precisely mirroring the activation profile observed in our *in vivo* scRNA-seq data (Fig. 3F, Fig. S2A). These results demonstrate that LECs are not merely passive conduits for waste but they act as an essential inflammatory intermediary that senses myelin debris and translates it into a potent biochemical signal for neutrophil activation and NETs formation. Given that the meninges also harbor other cell types, such as abundant border-associated macrophages (BAMs) and fibroblasts, these populations might also interact with neutrophils upon contact with myelin debris. To address this, we isolated primary BAMs and fibroblasts and co-cultured them individually with neutrophils. Immunofluorescence qualitative analysis and flow cytometric quantification of NETs revealed that following low-dose myelin treatment, only LECs possessed the capacity to stimulate increased NET release from neutrophils (Fig. 3G).

### LEC-derived Hexosylceramide (Hex1Cer) is the critical lipid mediator of NETs formation

**Fig. 4:**
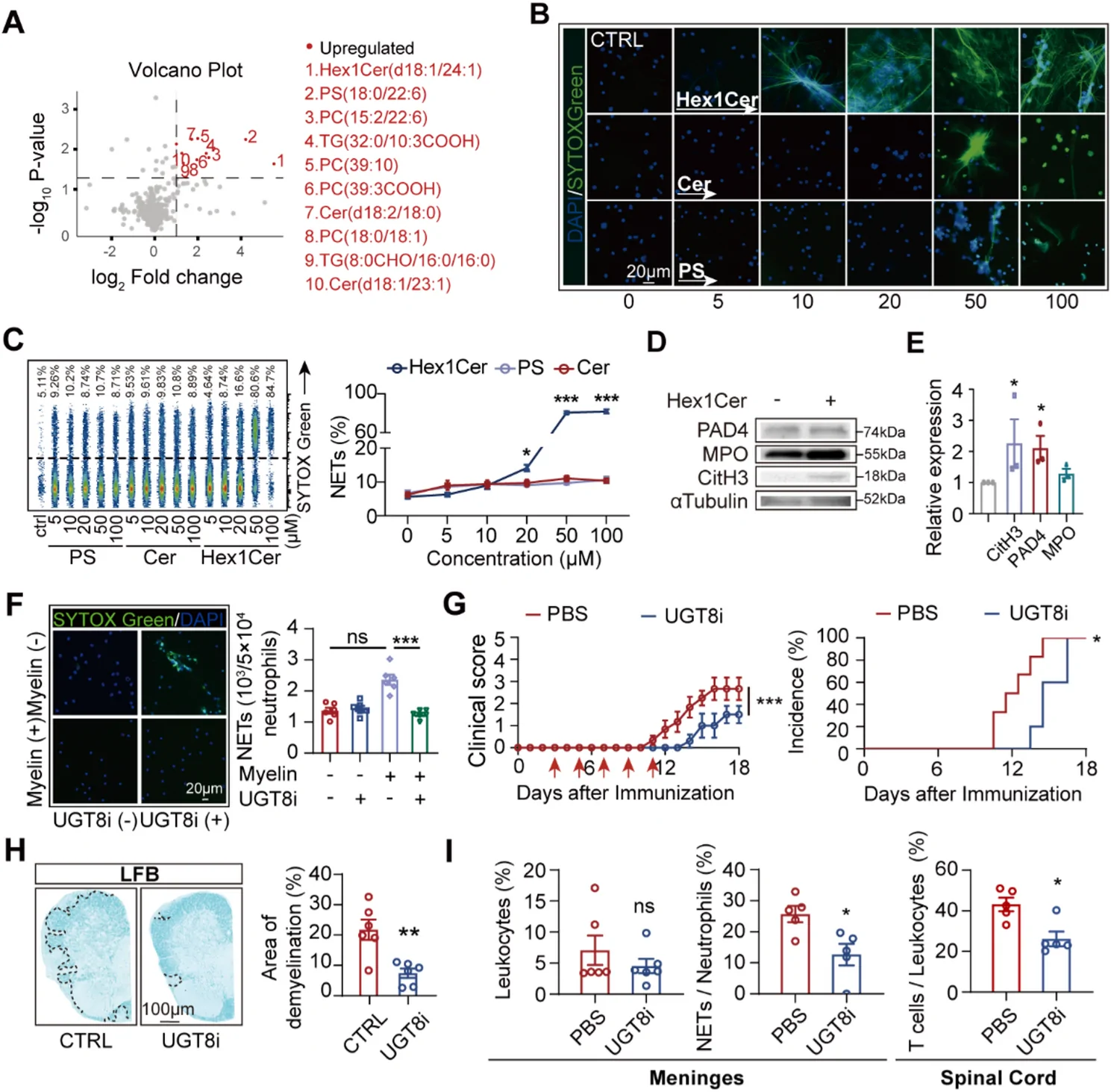
LEC-derived Hexosylceramide (Hex1Cer) is the critical lipid mediator of NETs formation. **A** Lipidomics analysis of LEC culture medium after myelin debris treatment (5 µg/ml, 24 h), followed by 4 h culture in 25% ECM+DMEM. Volcano plot displays 10 significantly altered lipids. Log2FoldChange > 1 and *P*.adjust value < 0.05. **B** IF of neutrophils treated with Hex1Cer, Cer, or PS. NETs formation (SYTOX Green, green) was dose-dependent only in Hex1Cer-treated groups. Scale bar = 20 μm. **C** Flow cytometry quantification of NETs. Pseudoplots and bar graphs confirm Hex1Cer-specific, dose-dependent NETs induction. Two-way *ANOVA*, N = 5, * *p* < 0.05, *** *p* < 0.001. **D**-**E** Neutrophils were treated with 20µM Hex1cer for 4 h and western blot suggested higher expression of protein PAD4 and CitH3. Experiment repeated for 3 times and quantitative analysis was done using One-way *ANOVA*. * *p* < 0.05. **F** UGT8i significantly inhibits myelin-pretreated mLEC-induced NET release in vitro. Scale bar = 20 μm. Two-way *ANOVA*, N = 5, *** *p* < 0.001, ns means no significant. **G** UGT8i was injected every 2 days after induction of EAE. Clinical score (left) and incidence (right) decreased with statistically significance after UGT8i treatment. Repeated measures two-way *ANOVA* or Student’s *t*-test, N = 6, * *p* < 0.05, *** *p* < 0.001. **H** LFB and analysis showed a reduced demyelination lesion in the lumbar spinal cord. One-way ANOVA, N = 5, ** *p* < 0.01. **I** Percentage of leukocytes (left), NETs (middle) in the meninges and T cells (right) in the spinal cord detected by flowcytometry showed a reduction in UGT8i group. N = 6, One-way *ANOVA*, * *p* < 0.05

To identify the specific factor secreted by myelin-primed LECs that triggers NETs formation, we performed Bulk RNA-sequencing on LECs treated with myelin debris. Gene Set Enrichment Analysis (GSEA) and GO enrichment revealed a significant upregulation of “Fatty acid metabolism” and slight change in the “Glycosphingolipid biosynthesis” pathways (Fig. S4E-F). Concurrently, mLECs and blood endothelial cells exhibited a higher pathway score for glucosylceramide synthesis compared with other cell types (Fig. S4G). mLECs showed a more oxidative condition after treated by myelin debris overnight (Fig.S4D). This metabolic shift prompted us to analyze the lipid composition of the LEC-conditioned media using unbiased comparative lipidomics. We identified a unique lipidomic signature in Myelin-LEC-CM, characterized by a specific and significant enrichment of Hexosylceramide (Hex1Cer) species (Fig. 4A). While Hex1Cer and related ceramides are known to accumulate in the CSF and lesions of MS patients, our data suggests that LECs actively contribute to this lipid pool upon sensing myelin debris [40, 41]. To validate the functional impact of this enrichment, we treated primary neutrophils with purified lipids. Purified Hex1Cer alone was sufficient to induce robust, dose-dependent NETs formation in vitro. In contrast, other abundant lipids identified in the CM, such as phosphatidylserine (PS) or Ceramide (Cer), failed to trigger NETs formation (Fig. 4B-C). Notably, while Ceramide induced cell death as indicated by SYTOX Green, it lacked the morphological and biochemical hallmarks of NETs, highlighting the specificity of Hex1Cer in this process. Mechanistically, Hex1Cer stimulation triggered the upregulation PAD4, the requisite enzyme for chromatin decondensation, and increased the expression of CitH3 (Fig. 4D-E). To confirm the in vivo pathogenicity of this axis, we treated EAE mice with an inhibitor of UGT8 (ceramide galactosyltransferase) every 2 days since dpi3, the rate-limiting enzyme for Hex1Cer synthesis. First, we verified in vitro that the drug significantly inhibited myelin-treated LEC-induced NET formation (Fig. 4F). Then we verified in vivo, since intrathecal injection directly delivers the drug into the cerebrospinal fluid bypassing the BBB, the systemic dose was converted to the local dose. We carefully calibrated the final intrathecal injection dose to be 0.02 mg per mouse, which ensures target inhibition in the CNS while minimizing peripheral toxicity. Local intrathecal delivery of the UGT8 inhibitor (UGT8i) significantly ameliorated EAE clinical scores, effectively phenocopying the results of lymphatic ablation (Fig. 4G-H). Although the transient nature of the inhibitor meant that mice eventually experienced disease onset, the blockade of Hex1Cer synthesis significantly reduced peak disease severity and was associated with a marked reduction in meningeal NETs formation (Fig. 4I). Furthermore, the recruitment of pathogenic T lymphocytes to the spinal cord was significantly attenuated. These results establish the LEC-derived Hex1Cer-NETs axis as a critical, druggable checkpoint that governs the transition of inflammation from the meninges to the spinal cord parenchyma.

### Meningeal NETs promote pathogenic T-cell retention via the SELPLG-SELL axis

**Fig. 5:**
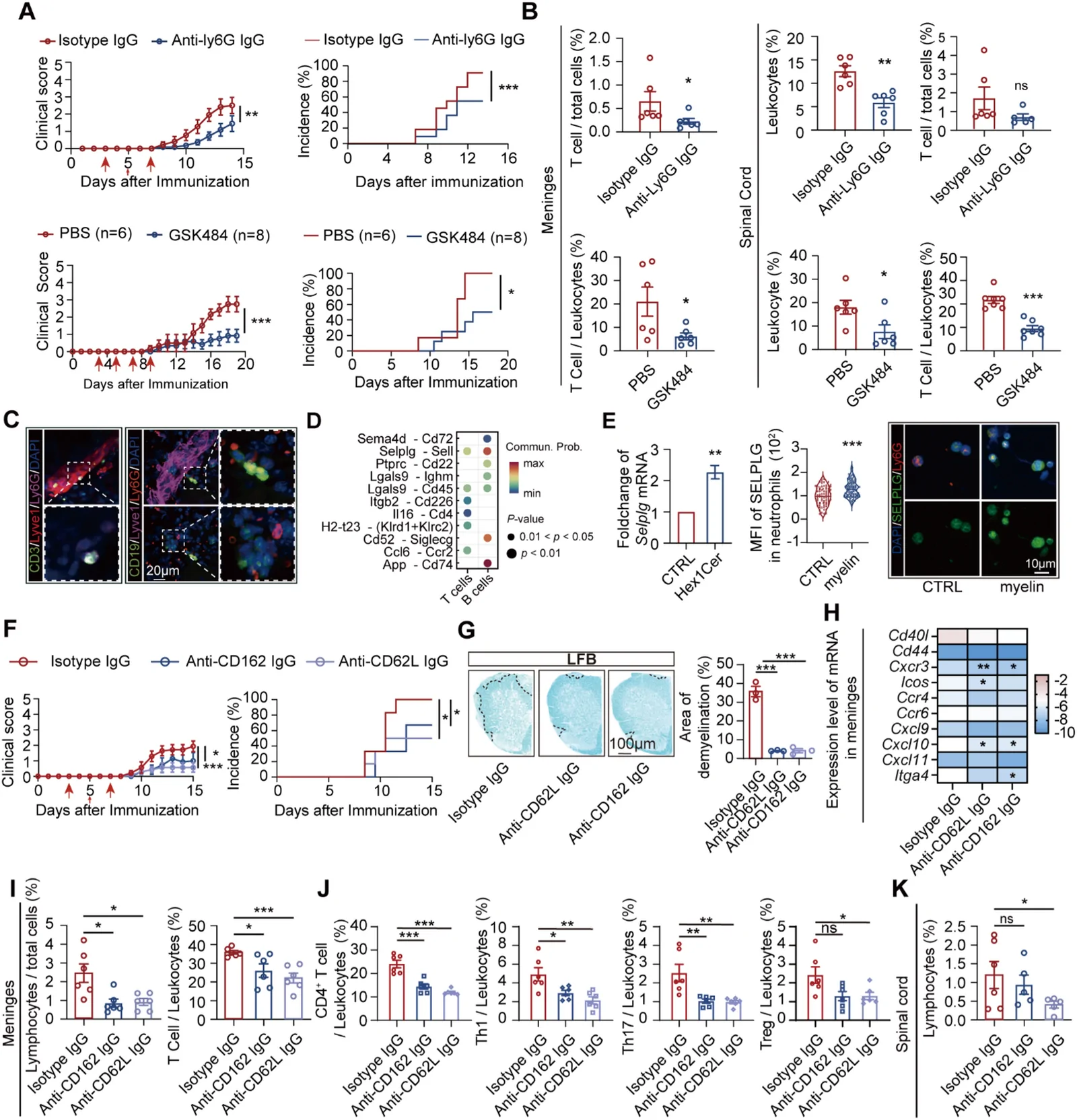
Meningeal NETs promote pathogenic T-cell retention via the SELPLG-SELL axis. **A** Daily EAE clinical scores and incidence. Anti-Ly6G and GSK484 significantly ameliorated disease. Repeated measures two-way *ANOVA*, N = 6, * *p* < 0.05, ** *p* < 0.01, *** *p* < 0.001. Student’s *t*-test for analyzing incidence. **B** Infiltration of leukocytes were analyzed by flowcytometry. In the meninges, CD45^+^ leukocytes and CD3^+^ T lymphocytes dropped while in the spinal cord T lymphocytes had a tendency of decrease. One-way *ANOVA*, N = 6, * p < 0.05. **C** IF confirming Ly6G^+^ neutrophil-CD19^+^ B cell interaction and neutrophil-T cell in EAE meninges. Scale bar = 5 or 20 μm. **D** Single cell analysis of meningeal cells suggested neutrophil had an enhanced interaction between B and T lymphocytes. Molecular pair Selplg-Sell was prominent in cellchat with both B and T lymphocytes. Log2FoldChange > 1 and *p*.adjust value < 0.05. **E** Hex1Cer-stimulated neutrophils showed elevated Selplg mRNA. One-way *ANOVA*, N = 3, ** *p* < 0.01. Coculture with myelin debris-primed LECs increased SELPLG (CD162/PSGL-1) expression in NETs-forming neutrophils (Ly6G, red; DAPI, blue). Analyzed by Student’s *t*-test, *** *p* < 0.001. **F** Daily EAE clinical scores and incidence. Anti-CD162 (SELPLG/PSGL-1) or anti-CD62L (SELL/L-selectin) slightly ameliorated disease. Repeated measures two-way *ANOVA*, N = 6, * *p* < 0.05, *** *p* < 0.001. **G** Luxol fast blue (LFB) staining indicated a significant reduction in the demyelinated area of EAE mice treated with anti-CD62L or anti-CD162 antibodies. One-way *ANOVA*, N = 3, *** *p *< 0.001. **H** Quantitative PCR (qPCR) analysis revealed downregulated mRNA expressions of *Cxcr3, Icos, *and *Cxcl10* in the anti-CD62L group, whereas the anti-CD162 group showed decreased expressions of *Cxcr3, Cxcl10*, and* Itga4* (N = 3 mice per group, with 3 technical replicates per animal). One-way *ANOVA*, N = 6, * *p* < 0.05, ** p < 0.01. **I**-**J** Flow cytometry demonstrated that both anti-CD62L and anti-CD162 treatments markedly reduced total lymphocyte and particularly T cell counts in the meninges of EAE mice. Within the T cell population, the proportions of CD4^+^ T cells, Th1, and Th17 subsets were significantly decreased, while Treg percentages remained unaltered. One-way *ANOVA*, N = 6, * *p* < 0.05, ** *p* < 0.01, *** *p* < 0.001. **K** In the spinal cord, total lymphocyte infiltration was substantially diminished in the anti-CD62L group, but showed no significant change in the anti-CD162 group. N = 6, One-way *ANOVA*, N = 6, * *p* < 0.05

Finally, we investigated the downstream consequences of meningeal NETs on the evolution of the autoimmune response. We first confirmed the inherent pathogenicity of this innate immune phase, both the systemic depletion of neutrophils (anti-Ly6G, 8.33 µg per mouse [42]) and the pharmacological inhibition of NETs formation (PAD4 inhibitor, GSK484, 0.133 mg/kg) significantly ameliorated EAE clinical severity (Fig. 5A, S5A). Notably, both interventions resulted in a profound reduction of T-cell infiltration into the meninges and spinal cord parenchyma, confirming that NETs formation is a requisite upstream event for the recruitment of the adaptive immune response (Fig. 5B). GSK484 significantly reduced the demyelination in LFB (Fig.S5B) and inhibited the infiltration of myeloid cells such as neutrophils in spinal cord and T cells in the blood and lymph nodes (Fig.S5C-F). Given the sticky physical properties of extruded chromatin, we hypothesized that NETs act as a physical scaffold to retain pathogenic lymphocytes within the meningeal space. High-resolution immunofluorescence (IF) of EAE meninges revealed that CD4^+^ T cells and CD19^+^ B cells were indeed physically colocalized within Ly6G^+^ structures (Fig. 5C, S6A). To define the molecular basis of this interaction, we revisited our scRNA-seq cellchat communication analysis, which predicted a high-probability interaction between neutrophils and lymphocytes via the *Selplg-Sell* axis (Fig. 5D). In this model, neutrophils express high levels of *Selplg* (coding PSGL-1/CD162), while T and B cells express its ligand, *Sell* (coding L-selectin/CD62L). Critically, we found that in vitro stimulation of neutrophils with Hex1Cer or myelin debris was sufficient to upregulate the surface expression of CD162 (SELPLG) (Fig. 5E). To test this functional axis in vivo, we administered either anti-CD162 (PSGL-1, 1.67 µg per mouse) or anti-CD62L (MEL14, 3.33 µg per mouse) blocking antibody to disrupt the NET-lymphocyte interface without depleting the neutrophils themselves. To evaluate* in vivo* target engagement, we assessed primary antibody binding following in vivo blockade via a competitive binding assay (as referenced by the manufacturer), confirming that the antibody effectively occupied both molecules in mice after EAE onset (Fig. S6B). This targeted blockade resulted in a significant improvement in clinical scores, less demyelination and a reduction in CNS T cell infiltration (Fig. 5F-G, I and K). To further investigate the mechanism by which NETs recruit lymphocytes, we examined the expression of various recruitment and chemotaxis related molecules in the meninges. Blockade of the SELPLG-SELL (CD162-CD62) axis downregulated meningeal expressions of *Cxcl10*, *Cxcr3*, and *Icos*, as well as *Itga4* encoding VLA4, a critical molecule for T and B cell trafficking into the central nervous system (Fig. 5H). Moreover, intercepting this axis also reduced the expression of certain lymphocyte chemotactic molecules in the spinal cord (Fig. S6C). Flow cytometry revealed a significant reduction in infiltrating leukocytes within both the meninges and spinal cord following pathway blockade (Fig. S6D, S6F). Further T cell subpopulation analysis indicated a marked decrease in the infiltration of both CD4^+^Tbet^+^ Th1 and CD4^+^IL17a^+^ Th17 cells, whereas Foxp3^+^ Treg cells showed no increasing trend (Fig. 5J, S6E). Together, these findings suggest that NETs drive EAE progression primarily by promoting T cell infiltration, particularly by expanding the populations of Th1 and Th17 cells, the two major pathogenic subsets. Collectively, our findings establish a new paradigm in neuroinflammation that meningeal lymphatic endothelial cells act as active orchestrators that sense early CNS damage. By initiating a pathogenic LEC-Hex1Cer-NETs axis, these vessels create a specialized physical niche that captures and amplifies the adaptive immune response, identifying multiple novel checkpoints for therapeutic intervention in MS.

### Intrathecally injection of Sivelestat ameliorated inflammatory demyelination in EAE

**Fig. 6:**
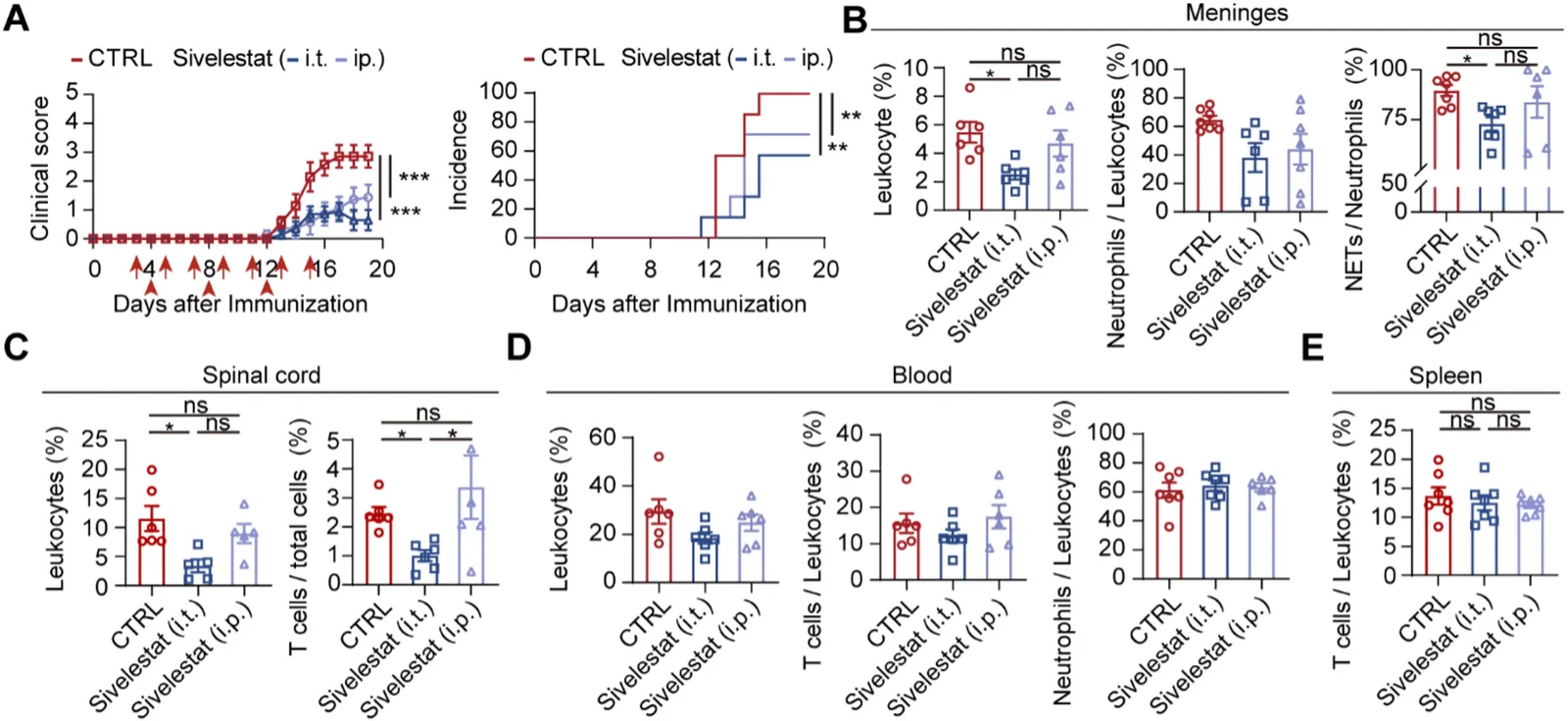
Local pharmacological inhibition of meningeal NETs formation by Sivelestat ameliorates EAE. **A** Sivelestat administration attenuates EAE clinical progression. Mean clinical scores (left) and cumulative disease incidence (right) in EAE mice treated with Sivelestat via intrathecal (i.t.) or intraperitoneal (i.p.) routes starting from the day of immunization. Red arrows showed the point of drug administration. *N* = 7 per group, Repeated measures two-way *ANOVA*, ** *p* < 0.01, *** *p* < 0.001. **B** Intrathecal Sivelestat suppresses meningeal NETs formation. Flow cytometric quantification of CD45^+^ total leukocytes, CD11b^+^Ly6G^+^ neutrophils, and CitH3^+^ NET structures in the meninges. Note the significant reduction of NETs in the i.t. group compared to vehicle controls. *N* = 6–8 per group, One-way *ANOVA*, * *p* < 0.05. **C** Reduced inflammatory infiltration in the spinal cord parenchyma. Absolute counts of CD45^+^ leukocytes and CD3^+^ T cells in the spinal cord demonstrate diminished neuroinflammation following local i.t. Sivelestat treatment. *N* = 6–8 per group, One-way *ANOVA*, * *p* < 0.05. **D** Maintenance of systemic neutrophil populations and T cells. Flow cytometric analysis of CD45^+^ leukocytes and CD3^+^ T cells in the peripheral blood (left), while circulating CD11b^+^Ly6G^+^ neutrophils (right) remain unaffected by i.t. treatment. *N* = 6–8 per group, One-way *ANOVA*. **E** Quantification of splenic CD3^+^T cells, indicating that local intervention in the meningeal niche didn't exert a systemic dampening effect on the adaptive immune response. *N* = 6–8 per group, One-way *ANOVA*

Given that meningeal NETs acts as a critical checkpoint for the initiation of demyelination, we sought to evaluate the therapeutic potential of blocking this process. We selected Sivelestat, a neutrophil elastase inhibitor clinically approved for acute lung injury [43], which effectively suppresses NETs generation. To distinguish between the roles of local meningeal NETs and systemic neutrophil activity, we compared intrathecal (i.t.) administration targeting the meningeal neutrophils with intraperitoneal (i.p.) administration targeting the systemic neutrophils at a dose of 12.5 mg/kg. Notably, the i.t. dose was set at 1/30th of the i.p. concentration. Treatment was initiated at the time of induction and maintained until the disease peak (Fig. 6A). Both routes significantly ameliorated EAE severity and lowered disease incidence. However, a striking divergence occurred upon treatment cessation, where the i.p. treated group showed signs of disease progression, whereas the i.t. treated group entered a stable remission phase, with several subjects becoming entirely asymptomatic (Fig. 6A). This prolonged protection at a significantly lower dose suggest that the meningeal niche is the earlier initial point of the disease, neutralizing it early prevents the establishment of a self-sustaining inflammatory cascade. Flow cytometric analysis revealed that while total neutrophil numbers in the meninges remained stable, consistent with our lymphatic inhibition data, the levels of CitH3^+^ NETs structures and total leukocyte counts were significantly reduced in the i.t. group (Fig. 6B). Most remarkably, the i.t. blockade of meningeal NETs formation led to a systemic reduction of T cell counts in the spinal cord but not in the peripheral blood or spleen (Fig. 6C-E). However, the i.p. blockade had a tendency of lower infiltration of leukocytes in the meninges, spinal cord and T cells in the spleen without significance. This indicates that the meninges are not just a local site of inflammation, but a primary amplification site that dictates the systemic autoimmune load. While the systemic i.p. treatment showed a similar trend during the onset stage, its effects waned rapidly upon the peak and withdrawal, likely because it failed to permanently disrupt the early pathological alterations to the CNS by adaptive immune cells. These findings underscore that neutrophil-mediated NETs formation within the meninges during the early phase is a more critical therapeutic target than later parenchymal demyelination. Collectively, our study identifies the meningeal lymphatic-NETs axis as a high-value clinical target, offering a novel conceptual framework for the timing and delivery of MS interventions.

## Discussion

This study identifies a pathogenic cascade in multiple sclerosis where the meningeal interface functions as a metabolic and immunological gateway. By integrating clinical neuroimaging with EAE mouse models, we show that meningeal inflammation is driven by a localized mLV-Hex1Cer-NETs. We claimed that the initial wave of meningeal neutrophils originated from the vertebral bone marrow, reaching the meninges via specialized osteal channels during the pre-clinical phase. This local recruitment bypassed systemic circulation, suggesting that hematopoietic changes in CNS-adjacent marrow niches precede neuroinflammation. Drawing on MRI protocols used for multiple myeloma [44], we propose that 7.0T MRI radiomics [45] of these marrow compartments could serve as a clue to predict relapses before T cells infiltrate the spinal cord. Our findings diverge slightly yet significantly from the conventional perspective regarding the initial stages of neuroinflammation (EAE clinical score no less than 1). While previous studies have suggested that meningeal neutrophil infiltration arose from both the systemic circulation and CNS-adjacent bone marrow niches [25, 46], which is consistent with our own observational data, we demonstrated a functional hierarchy between these populations. Specifically, by selectively depleting vertebral bone marrow, we observed a significant amelioration of EAE severity. However, several caveats must be noted when interpreting these findings though the irradiation experiment confirmed the part of the neutrophils were derived from vertebral bone marrow. The pathogenesis of demyelination is inherently a multicellular orchestrated process in which both resident and infiltrating immune cells are intricately involved. Although our bone marrow chimera and targeting strategies demonstrate a pivotal role for spinal bone marrow-derived cells, they do not strictly rule out potential secondary effects of irradiation on CNS resident populations, such as parenchymal microglia. Though currently, it is acknowledged that irradiation at this dosage can induce the upregulation of central pro-inflammatory cytokines and the activation of resident microglia [47]. Theoretically, such irradiation-induced microglial alterations would be expected to exacerbate disease onset and progression. Intriguingly, however, our findings demonstrated a significant attenuation of clinical symptoms following bone marrow ablation. This apparent paradox warrants further investigation. At the parenchymal-meningeal interface, border-associated macrophages (BAMs) participate in neuroimmune surveillance and function as local antigen-presenting cells to amplify T cell driven inflammation [48, 49]. Furthermore, infiltrating autoreactive CD4⁺ T cells, particularly Th1 and Th17 subsets, are reactivated by resident myeloid cells and mediate oligodendrocyte injury via IFN-γ and IL-17 signaling [50]. Consistent with this complex cellular interplay, bone marrow ablation broadly downregulated multiple immune subsets, including both neutrophils and T cells. We cannot exclude the possibility that unmeasured cell populations, such as eosinophils, mast cells, or dendritic cells, were similarly altered.

We observed that neutrophils exhibit a distinct propensity to undergo NETs formation within the meninges during the early stage of EAE. This meningeal NETs formation has also been documented in other pathological conditions, such as *Streptococcus pneumoniae* meningitis and subarachnoid hemorrhage [51, 52]. Interestingly, in *Streptococcus* meningitis, the massive accumulation of NETs obstructs cerebrospinal fluid (CSF) drainage pathways, thereby mediating the development of brain edema and reduce bacterial clearance. These disparate findings suggest that the composition and release modalities of meningeal NETs are not uniform across different diseases, underscoring a high degree of NET heterogeneity. Distinct from the conventional lytic NETosis that typically yields massive, sticky reticular webs, hexosylceramide-induced NETs instead exhibit a specialized phenotype optimized for the chemotactic recruitment of other immune cells. This divergence also implies that the physiological function of NETs may vary depending on the local antigen load, specifically between the early disease phase with trace myelin debris and the peak phase characterized by the drainage of overwhelming amounts of myelin fragments. Whether NETs generated at the peak of EAE produce a similar obstructive phenomenon warrants further investigation.

Although our findings reveal that mLVs participate in draining myelin debris, a detailed quantitative analysis of post-demyelination myelin metabolic kinetics was beyond the scope of this study, which primarily focused on the immune cascades elicited by drained myelin components. Considering that precise clearance pathways are vital for understanding demyelination and subsequent remyelination, whether mLVs constitute the principal or unique route for myelin clearance requires more extensive exploration. Single-cell transcriptomic analyses revealed that enzymes involved in hexosylceramide synthesis and metabolism are broadly expressed across various resident cell types, including blood endothelial cells and fibroblasts. Fundamentally, these enzymes are essential for basal sphingolipid homeostasis, maintaining cell membrane integrity, and supporting myelination. However, our focus on lymphatic endothelial cells in driving NETs formation is anchored on both spatial and functional evidence. Spatially, immunofluorescence staining demonstrated that myelin debris is cleared via mLVs in the early stage, with NETs formation preferentially clustering within and around the mLV microenvironment during early EAE. Functionally, our in vitro screening showed that myelin-pretreated resident populations, such as border-associated macrophages and abundant meningeal fibroblasts, failed to trigger NETs formation in co-cultured neutrophils. Thus, while other cell types possess the transcriptomic machinery for Hex1Cer synthesis, where their precise pathological roles during neuroinflammation warrant further exploration, our data establish that myelin-activated LECs specifically orchestrate the Hex1Cer-dependent induction of meningeal NETs at disease onset.

This anatomical recruitment is regulated by mLECs, which act as metabolic sensors for myelin debris. Following the drainage of myelin fragments from nascent CNS lesions, mLECs underwent metabolic reprogramming, resulting in the synthesis and secretion of Hex1Cer. Hex1Cer subsequently triggered NETs formation in the resident neutrophils. Notably, VEGFR3 inhibition prevented NETs formation without reducing total neutrophil counts at the peak stage of EAE. These data support a two-step activation model, while multiple pathways recruit neutrophils to the meninges, their functional transition into a pathogenic, NET-forming state requires licensing by the lymphatic endothelium. Thus, the mLEC-Hex1Cer axis represents a specific checkpoint where tissue damage is converted into an innate immune response. Previous clinical observations have indicated elevated levels of ceramide and Hex1Cer in the cerebrospinal fluid of patients with multiple sclerosis compared to healthy controls [40], a finding that aligns with our current results. While such lipid accumulation is partially attributable to the metabolic breakdown of myelin within demyelinating lesions [41], our study provides a novel mechanistic perspective on the possibility how these metabolites drive disease relapse and progression. Due to the limited availability of clinical specimens at our center, a broader characterization of the correlation between Hex1Cer concentrations and clinical parameters in MS patients remains to be conducted. While previous studies have reported that VEGFR3 inhibition fails to ameliorate EAE severity via the reduction of mLV density [35], our quantitative flow cytometric analysis revealed a slight but significant decrease in mLEC counts detected by flowcytometry. We thought that this discrepancy might arise not only from the methods, but also from the multiple roles of VEGFR3 in mLV function. Our findings suggest that VEGFR3 signaling may be essential for drainage efficiency and capacity to sense and process drained cargo, such as myelin debris, however this needs more solid evidence.

Notably, while most research on meninges focuses on T and B cell interactions and the formation of ectopic lymphoid structures [50, 53], our work shifts the emphasis toward the pivotal role of innate immune cells in orchestrating this pathogenic gateway. The interaction between mLVs and neutrophils established a physical scaffold for adaptive immune infiltration. We found that NETs acted as a molecular bridge, sequestering encephalitogenic T and B cells within the meningeal space via the SELPLG-SELL axis. This pathological stasis impeded immune cell clearance and promoted local activation and subsequent spinal cord invasion. This feed-forward loop, where myelin activated mLVs trigger NET scaffolds that facilitate T cell recruitment, explains why meningeal enhancement correlates with disability progression. Furthermore, low-dose intrathecal Sivelestat (at 1/30th of the systemic dose) effectively suppressed the disease, confirming the meninges as the primary inductive hub. Targeting this gateway may dismantle the inflammatory scaffold while potentially mitigating the severity of systemic immunosuppression compared to traditional systemic delivery, although partial systemic immunomodulatory effects cannot be entirely excluded. This study has several limitations. First, while myelin debris triggers mLEC activation, the primary etiology initiating the initial demyelination remains unknown. Second, the role of mLVs may be context dependent, our EAE data suggest a switch from a pro-inflammatory state in the acute phase to a potentially pro-regenerative role in the chronic stage [54]. Future work with inducible genetic tools is needed to define these temporal windows. Third, while NETs trap lymphocytes, their role in maintaining therapy resistant ectopic lymphoid structures requires further study. Finally, the scarcity of human post-mortem meningeal tissue from subclinical stages limits the direct mapping of the Hex1Cer pathway in patients, though our MRI data and Sivelestat experiments provide translational evidence.

In conclusion, we have mapped a pathogenic axis involving mLV-mediated metabolic reprogramming, marrow bone-to-meninges mobilization of neutrophils, and NETs dependent lymphocyte recruitment. This work orchestrates targetable checkpoints, including UGT8-mediated lipid synthesis and the SELPLG-SELL adhesion axis. Disrupting the innate-to-adaptive transition at the meningeal gateway offers a strategy to intercept MS before progressive neurodegeneration occurs.

## Materials and methods

### Human material

The current research was approved by the ethics of committee of the Third Affiliated Hospital of Sun Yat-sen University. Patients were included if they met the 2024 Revisions of the McDonald Diagnostic Criteria for relapsing-remitting multiple sclerosis (RRMS) and were admitted during an acute relapse phase (defined as clinical neurological symptoms persisting for greater than 24 h) [55]. Crucially, to prevent potential confounding artifacts from treatment or secondary systemic inflammation, all evaluations were performed upon admission prior to the initiation of high-dose corticosteroid treatment in this relapse. Patients were strictly excluded if they presented with confounding comorbidities, including intracranial infections, stroke, alternate neuroinflammatory disorders, or systemic autoimmune diseases (such as systemic lupus erythematosus). Patient was selected and expanded disability scale score (EDSS) was assessed during admission by one investigator in double blind pattern. Additionally, to evaluate meningeal enhancement, gadolinium-postcontrast magnetic resonance imaging protocols, specifically featuring T2-weighted postcontrast turbo spin-echo (TSE) dark-fluid fluid-attenuated inversion recovery (FLAIR) sequences or T1-weighted postcontrast TSE sequences, were acquired shortly after admission. Comprehensive demographic parameters, including age, biological sex, disease duration, have now been fully itemized in the Supplementary Table 1.

### Induction of murine experimental encephalomyelitis model (EAE) 

EAE was induced as reported [56] in 8–10 weeks old female C57BL/6 mice purchased from Guangzhou Ruige Biological Techonlogy Co.,Ltd. The antigen emulsion was prepared by mixing MOG_35–55_ (Scilight Biotechnology, SL2134-2) in PBS with an equal volume of complete Freund’s adjuvant (Chondrex, 7023). Each C57BL/6 mouse was immunized via subcutaneous injection of 0.2 mL (containing 200ng MOG_35−55_) of the antigen emulsion into its back. Pertussis toxin (200 ng, Merck Milliepore) was administered intraperitoneally on days 0 and 2. During the experiment, mice were observed daily in a double-blind manner, and clinical scores were assigned based on the following criteria: 0 = no clinical signs;1 = paralyzed tail; 2 = hind limb paresis; 3 = both hind limbs paralyzed; 4 = forelimbs paralyzed; 5 = moribund.

(1) Naïve Stage: Age-matched healthy female mice without immunization, analyzed at day 0. (2) Pre-clinical stage: Mice started EAE induction but without symptoms sacrificed at dpi7. (3) Onset Stage: Defined by the initial manifestation of neurological symptoms (clinical score = 1), typically occurring around dpi 12; tissues were harvested precisely at the appearance of symptoms. (4) Peak Stage: Defined by maximum neurological deficit and disease severity (clinical score ≥ 3), consistently observed between dpi 15 - 18. (5) Chronic stage: mice at dpi45.

### Localized bone marrow depletion via fractionated irradiation 

To selectively deplete hematopoietic progenitors within the skull and vertebral bone marrow niches, adult wild-type mice were subjected to a fractionated, localized X-ray irradiation protocol [37]. Briefly, mice were anesthetized with an intraperitoneal (i.p.) injection of 1.25% tribromoethanol (Merklin, T831042, 250 mg/kg body weight). To ensure the survival of the mice and the integrity of the peripheral immune system (e.g., femur bone marrow and spleen), mice were immobilized within a custom-engineered lead shielding chamber. The shield was specifically designed with precision apertures to expose only the skull and the vertebral column while providing comprehensive protection (> 95% attenuation) to the thorax, abdomen, and limbs. The irradiation was delivered using an X-ray irradiator (RS-2000, Rad Source Technologies) at a total dose of 11 Gy, administered in two equal fractions of 5.5 Gy. A 6-hour interval was maintained between the two doses to minimize potential non-target toxicity and gastrointestinal distress. Following the second dose, mice were monitored for recovery in a temperature-controlled environment with ad libitum access to food and water. On the second day, mice were induced EAE model.

### Vertebral and cerebral meninges separation

Mice were anesthetized by 1.25% tribromoethyl alcohol. Cut along the spine, separate it from the brain at the 1st vertebrae, carefully remove the muscle adherent to the bone. Put the spine under the microscope with ventral side up. Drop small amount PBS on the spine. Take care cutting along pedicles bilaterally and remove vertebra body so to avoid destroying spinal cord. Repeat the same work until complete spinal cord was exposed. Slightly detach spinal cord from the remaining bone and keep the meninges complete around the spinal cord. At last, peel the meninges from the spinal cord to perform the following immunofluorescence or flow cytometric fluorescence. As for brain, mysenchyma was separated from the cranium and meninges which is adjacent to the skull bone was removed with fine scissors in the PBS.

### Intra-spinal cord (i.sc.) stereotaxical injection and local demyelination model induction

Mice were anesthetized with 1.25% tribromoethanol before being positioned in a stereotaxic frame (RWD Life Science Co., LTD, 68045). Following a skin incision, a small craniectomy was performed using a micro handheld cranial drill (RWD Life Science Co., LTD, 78001), and injections were administered via a 5µL Hamilton syringe in a speed of 0.5 µl/min. To prevent backflow, the capillary remained in place for five minutes. To investigate the drainage route of myelin debris, we labeled the white mater of spinal cord using the lipid dye 1,1’-dioctadecyl-3,3,3’,3’-tetramethylindocarbocyanine perchlorate (DiI, Invitrogen, D282) and induced demyelination with lysophosphatidylcholine (LPC, MCE, HY-139414). Dil (i.c., 100 µg/mL, 5 µL, 1uL/min) was stereotaxically injected into the lumbar level of spinal cord in naïve WT mice (8–12 weeks old) to label myelin. After a 4-day period for clearance of excess DiI, LPC was stereotaxically injected in situ to induce demyelination (i.c., 5 mg/mL, 1.5 µL, 0.5µL/min). Mice were sacrificed 1 day, 3 days, 7 days post-LPC injection for analysis of myelin debris drainage. The similar method was reported before in the study of brain [57].

### Intrathecal injection

Mice were anesthetized with 1.25% tribromoethyl alcohol (Merklin, T831042). Each mouse was positioned ventral side down over a 5 mL syringe to widen the intervertebral space. Using a Hamilton syringe fitted with an insulin needle, 5 µL of drug or PBS was administered at the L5/6 level. Penetration of the dura was confirmed by a tail flick reflex elicited by nerve root stimulation. The injection was delivered over a period of more than 5 min. The needle was left in place for an additional 3–5 min to prevent backflow.

Drug:

- VEGFR3IN1 (MedChemExpress (MCE), Cat. No. HY-112211)
- Sivelestat (MedChemExpress (MCE), CAS. No. :127373-66-4, HY-17443)
- UGT8-IN-1 (MedChemExpress (MCE), CAS No. :2414349-93-0, HY-131703)
- GSK484 (MedChemExpress (MCE), HY-123606)
- Anti-Mouse Ly6G In-Vivo (Selleck, A2158)
- Anti-Mouse L-Selectin/CD62L Antibody (Mel-14) (MedChemExpress (MCE), HY-P990201)
- Anti-Mouse PSGL-1/CD162 Antibody (4RA10) (MedChemExpress (MCE), HY-P990166)

The administration points were showed with red arrows in every clinical score figure of treatment.

The administered doses of all pharmacological agents were primarily calculated based on the manufacturers’ recommended specifications and calculated for intrathecal injection with further reference to previous literature [58].

### Mouse bone marrow-derived neutrophil (BMDN) isolation and culture

Neutrophils were isolated from bone marrow of 8-week‐old male mice by Percoll (GE Healthcare) gradient centrifugation according to published methodology [59]. Mice were euthanized by cervical dislocation. The hind legs were harvested, and the ankle and knee joints were disarticulated. Muscles were carefully removed to isolate the femurs and tibias. The bones were immersed in 75% ethanol for 2 min and transferred into a biological safety cabinet. All subsequent steps were performed under sterile conditions. After washing with PBS, the bones were placed in a petri dish. A 10 mL syringe barrel fitted with a 1 mL syringe needle was used to flush out the bone marrow. Both ends of each bone were cut, and the syringe was inserted into the marrow cavity to flush with PBS. The flushed marrow was collected in a 15 mL centrifuge tube and dispersed in PBS. The cell suspension was centrifuged at 500 × g for 5 min. The pellet was resuspended and layered onto a discontinuous Percoll gradient (Cytiva, 17089109-1) of 65% and 58.5%, followed by centrifugation at 800 × g for 18 min (acceleration 3, deceleration 3). The second layer from the top, containing neutrophils, was collected and washed once with PBS. Neutrophils were cultured in RPMI 1640 medium supplemented with 10% FBS (Vivacell, C04001-500) and 1% Penicillin-Streptomycin (Invitrogen, 15070063).

### Mouse bone marrow-derived macrophage isolation and culture

Bone marrow single-cell suspensions were prepared following the same protocol described for BMDN isolation. Briefly, the harvested bone marrow cells were filtered through a 70 μm nylon cell strainer to remove tissue debris. To eliminate erythrocytes, the cell suspension was incubated with 1× ACK lysis buffer for 2–3 min at room temperature. The lysis reaction was subsequently neutralized by adding an excess volume of phosphate-buffered saline (PBS), followed by centrifugation at 500 g for 5 min. The resulting cell was resuspended and seeded into non-adherent 15 cm culture dishes. The cells were cultured in DMEM + 10%FBS and 1% penicillin-streptomycin (P/S) + 10% L929 condition medium for 7 days for harvesting and further use.

### Mouse meningeal fibroblast isolation and culture

The isolation of primary meningeal fibroblasts was performed in accordance with established protocols [60–62]. Briefly, the meninges were carefully dissected and peeled from the cerebral hemispheres of postnatal day 0 (P0) mouse pups. The harvested meningeal tissues were enzymatically digested with 0.25% trypsin + 1% collagenase IV in a 37 °C water bath for 30 min. The digestion process was neutralized by adding an equal volume of complete medium containing 10% FBS, and the mixture was passed through a 70 μm nylon cell strainer to remove undigested tissue chunks. The resulting single-cell suspension was centrifuged at 500 g for 5 min to pellet the meningeal fibroblasts. The cells were subsequently resuspended and cultivated in DMEM + 10%FBS and 1% penicillin-streptomycin (P/S) until they reached confluence, after which they were passaged for downstream experimental utilization.

### Myelin debris extraction and purification

Mice were euthanized via decapitation. Following surface sterilization of the cranium with 75% ethanol, intact brains were rapidly dissected in a laminar flow hood and placed into sterile culture dishes containing ice-cold phosphate-buffered saline (PBS). Brain tissues were transferred to clean culture dishes and minced into fine fragments using sterile micro-scissors. Enzymatic digestion was initiated by adding 0.25% trypsin-EDTA solution at a ratio of 1 mL per brain, followed by incubation at 37 °C for 30 min in a humidified incubator. Post digestion, the tissue fragments were mechanically dissociated into a homogeneous suspension via serial passage through sterile micropipette tips with decreasing orifice diameters. The resulting homogenate was passed through a 40–70 μm nylon cell strainer into a 50-mL centrifuge tube. To maintain a strict final concentration of 30% Percoll during filtration, each single enrichment system was adjusted to a total volume consisting of 2 mL brain homogenate, 3 mL 100% Percoll, and 5 mL 0.15 M NaCl. A discontinuous density gradient was established in a 15mL centrifuge tube by underlaying 2–3 mL of 70% Percoll beneath the filtered 30% Percoll brain homogenate using a sterile Pasteur pipette. Centrifugation was performed at 800 g for 15 min above 18 °C, with acceleration and deceleration rates set to a gentle profile (Accel: 3, Decel: 3) to protect the gradient interface. Following centrifugation, the crude myelin fraction accumulating at the topmost layer was carefully collected using a micropipette, strictly avoiding disturbance of the underlying aqueous phases. The collected myelin was transferred to a fresh 15 mL tube and washed twice with PBS. For every 2–3 pooled brains, the pellet was resuspended in 500 µL PBS and pelleted at 800 g for 5 min. The final purified myelin pellet was aliquoted into sterile 200 µL microcentrifuge tubes. Protein concentration was determined utilizing a standard Bicinchoninic Acid (BCA) protein assay kit with a 2µL representative sample. Aliquots were stored at -20 °C for subsequent downstream functional assays.

### Neutrophil extracellular traps (NETs) induction

After isolation, neutrophils were allowed to sediment onto PEI-precoated plates (Proteintech, PR40001) for 20 min. The culture medium was then removed, and cells were incubated in fresh medium containing the respective treatments for 4 h to induce NETs formation. Following incubation, the medium was aspirated and the wells were gently washed with cold PBS. Neutrophils were fixed with 4% paraformaldehyde (PFA) for 20 min at room temperature and subsequently prepared for immunofluorescence (IF) staining.

### Neutrophil coculture with lymphatic endothelial cells (LECs)

LECs were pre-cultured in the upper chambers of 24-well Transwell plates with or without myelin debris. After isolation, neutrophils were seeded into the lower chambers of the plates and allowed to sediment for 30 min. The LECs, along with the Transwell inserts, were then washed, positioned above the neutrophil layer, and co-cultured for 4 h prior to harvest.

### Flow cytometry

Lymphatic endothelial cells or mixed cell populations from mice were fixed with fixation buffer (Invitrogen) for 20–30 min at room temperature. After one wash with PBS, the cells were resuspended in PBS. For some comparative analyses, cells from different experimental groups were stained simultaneously using the same antibody panel at 4 °C overnight in the dark. Anti-CitH3 (1:500) antibody was used in 4 ℃ overnight as primary antibody as IF and secondary antibody conjuncated with 488 was used for 1 h in room temperature. All fluorescent antibodies used are listed below. Prior to analysis, all cells were washed once with PBS and resuspended in 100 µL PBS. Samples were acquired on a flow cytometer and analyzed using NovoExpress (v1.5.6) and FlowJo (v10.8.1). Gating strategies are provided in the Supplementary Figure.

The antibody panel included: Zombie Aqua™ Fixable Viability Dye (BV510), BV421 anti-CD45 and PerCP-Cy5.5 anti-CD45 for total leukocytes; FITC anti-CD3 for T lymphocytes; APC-Cy7 anti-B220 for B lymphocytes; PE-Cy7 anti-CD11b for total myeloid cells; APC anti-F4/80 for macrophages; and APC anti-Ly6G or APC-Cy7 anti-Ly6G for neutrophils. All antibodies were purchased from BioLegend and used at a 1:400 dilution. Neutrophil extracellular traps (NETs) were detected using SYTOX Green (1:30,000, Invitrogen, S7020) or an anti-citrullinated histone H3 (R2 + R8 + R17) antibody (1:500, Abcam, ab281584). Other antibodies included anti-mouse CD62L antibody (1:500, Proteintech, 26477-1-AP) and anti-mouse CD162/SELPLG antibody (1:500, Affinity Biosciences, DF6536).

### RNA retraction and Reverse transcription-quantitative PCR

RNA was retracted with RNA Quick Purification kit (ES Science, RN001) and concentration was measured with Nanodrop. RNA was reversed to cDNA for detecting the expression of certain DNA segment. The thermocycling conditions involved an initial step at 95 °C for 15 min, followed by 40 cycles at 94 °C for 15 s, 55 °C for 30 s, and 70 °C for 30 s. Following the PCR procedure, the relative expression levels were calculated using the 2 − ΔΔCq method. Experiment repeated thrice. All the primers were listed here. *Selplg* primer forward: CAGAGACCTCAAAACCAGCACC. *Selplg* primer reverse: GGTAGGTTCTGTGGAAGGGACT. *Cd44* primer forward: CGGAACCACAGCCTCCTTTCAA, *Cd44* primer reverse: TGCCATCCGTTCTGAAACCACG; *Cd40L* primer forward: GAACTGTGAGGAGATGAGAAGGC, *Cd40L* primer reverse: TGGCTTCGCTTACAACGTGTGC; *Icos* primer forward: GCAGTTTCGTTGTGGTACTCC, *Icos* primer reverse: TGTGTTGACTGCCGCCATGAAC; *Cxcr3* primer forward: TACGATCAGCGCCTCAATGCCA, *Cxcr3* primer reverse: AGCAGGAAACCAGCCACTAGCT; *Itga4* primer forward: GCAAAGAGGTCCCAGGCTACAT, *Itga4* primer reverse: CCTGTAATCACGTCAGAAGTCCC; *Ccr6* primer forward: ACAGAGCCATCCGAGTCGTGAT, *Ccr6* primer reverse: CTGGTGTAGGCGAGGACTTTCT; *Ccr4* primer forward: GGACTAGGTCTGTGCAAGATCG, *Ccr4* primer reverse: TGCCTTCAAGGAGAATACCGCG; *Cxcl9* primer forward: CCTAGTGATAAGGAATGCACGATG, *Cxcl9* primer reverse: CTAGGCAGGTTTGATCTCCGTTC; *Cxcl10* primer forward: ATCATCCCTGCGAGCCTATCCT, *Cxcl10* primer reverse: GACCTTTTTTGGCTAAACGCTTTC; *Cxcl11* primer forward: CCGAGTAACGGCTGCGACAAAG, *Cxcl11* primer reverse: CCTGCATTATGAGGCGAGCTTG.

### Western blotting

Cellular proteins were extracted using RIPA lysis buffer (Solarbio, R0010) supplemented with 1% PMSF (Beyotime, ST506) and phosphatase inhibitors (MCE, HY-K0021). Protein concentrations were determined by BCA assay (Beyotime), and samples were denatured by boiling in SDS loading buffer. Equal amounts (10 µg) of protein were loaded onto 10% acrylamide gels. Electrophoresis was performed at 90 V until samples condensed in the stacking gel, followed by separation at 140 V in the resolving gel. Proteins were then transferred to a PVDF membrane at 300 mA for 1.5 h. The membrane was blocked with 5% BSA in TBST for 1 h and subsequently incubated with primary antibody at 4 °C overnight. After washing, the membrane was incubated with a secondary antibody, either overnight at 4 °C or for 1 h at room temperature. Protein bands were visualized using an ECL (Mei5bio, MF074) detection system, with exposure times ranging from seconds to minutes. Signal intensities were analyzed using ImageJ software.

### Enzyme-Linked Immunosorbnent Assay (ELISA)

Culture media were centrifuged in 1000 g, 10 min and supernant were extracted to be test. Using the ELISA kit (MEIMIAN).

### Immunofluorescence (IF)

Spinal cords were dissected and fixed overnight in 4% PFA. Tissue was sectioned at a thickness of 20 μm using a cryostat. Meninges and spinal cord sections were stored in a cryoprotectant solution composed of phosphate buffer, glycerol, and ethylene glycol (450:250:300) at -20 °C. For staining, sections were washed three times for 5 min each in PBS and permeabilized with 0.25% PBST for 15 min at room temperature (RT). After washing, samples were blocked with 3% BSA in 0.03% PBST for 1 h. They were then incubated with primary antibodies overnight at 4 °C, followed by incubation with fluorescently labeled secondary antibodies for 1 h at RT. Nuclei were counterstained with DAPI, and slides were imaged using a Nikon or Leica confocal microscope.The following primary antibodies were used: anti-LYVE1 (1:500, Abcam) and anti-dMBP (1:3000, Merck).

### Tyramide signal amplification (TSA)

Permeabilization and blocking steps were performed as described for the standard immunofluorescence protocol. Primary antibodies were diluted at higher multiples and applied overnight at 4 °C. Following primary antibody incubation, sections were sequentially incubated with an HRP-conjugated secondary antibody and then with a tyramide-fluorophore conjugate, each for 10 min. Antigen retrieval was performed by boiling sections in 2% sodium citrate buffer (pH 6.0) at 65–85 °C for 30 min. After cooling, sections were re-blocked with serum before subsequent antibody incubations, which followed the standard immunofluorescence procedure.The following antibodies were used for TSA: anti-dMBP (1:9000, Merck), anti–citrullinated histone H3 (R2 + R8 + R17) (1:1500, Abcam, ab281584), anti-MPO antibody conjugated to 488 (Abcam), anti-mouse SELPLG antibody (1:1500, Affinity Bioscience, DF6536), anti-mouse L-selectin/CD62L (1:500, Proteintech, 26477-1-AP), and anti-mouse Ly6G antibody (1:500, Proteintech, 65140-1-Ig).

### RNAseq analysis

Dataset GSE168202 was downloaded from Gene Expression Omnibus (GEO) database [35]. Downstream analysis ran in the Seurat v3 R-based package. Matrix form spinal dura statistics were loaded to create 3 objects in Seurat v3. According to the analysis of primary owners of the dataset, mitochondria gene no more than 20% and gene count between 200 and 6000 were retained to perform further analysis. The data was then normalized(‘LogNormalize’) and top 2000 variable genes were sought out (FindVariableFeatures, method ‘vst’) to harmonize the data from different mouse (IntegrateData). Based on the scaled data, linear dimension reduction (principal component analysis, PCA) was performed and top10 PCA was loaded into clustering. By using the function ‘FindNeighbors (resolution = 0.5)’ in the Seurat package, all nuclei were assigned into 18 groups. Marker genes were sorted out by ‘FindAllMarkers, min.pct = 0.1’ and name every cluster by their main features. Then celldex and SingleR packages were loaded in the work space assisting us to rename clusters. Cluster visualization was calculated with the uniform manifold approximation and projection (UMAP), group by ‘orig.ident’. Enrichment analysis was performed by the Clusterprofiler package and results were visualized in the dotplot function (Cutoff value, log2FoldChange > 1 and *p*-value < 0.05). Cell cell communication was run by Cellchat package.

### Bulk RNA-seq analysis

Raw sequencing data were processed and analyzed in RStudio (v4.2.2). Gene expression levels, measured in FPKM (gene_fpkm), were compared between control and treatment groups using the DESeq2 package. Differential expression analysis was performed based on the gene_fpkm_group factor. Genes were annotated and classified according to their log2FoldChange (log2FC) and adjusted p-value (padj). The thresholds for defining significant differential expression were set as |log2FC| ≥ 1 and *padj* < 0.05 (up-regulated, log2FC ≥ 1; down-regulated, log2FC ≤ -1). Subsequently, these differentially expressed genes (DEGs) were subjected to enrichment analysis using the ClusterProfiler package to identify significantly altered biological pathways. This bulk RNA-seq analysis provides evidence for transcriptome-wide variations in response to the treatment.

### Lipidomics

Lymphatic endothelial cells were grouped by with or without myelin debris treatment. After 24 h cell culture medium was collected in the 15 ml centrifuge tube and centrifuged for 1000 g, 10 min, 4℃. The supernant was divided equally in the 2 ml cryogenic vials and quick frozen in the liquid nitrogen for several minutes until the samples completely turned into solid. The samples were stored in the − 80℃ refrigerator before uploading in the machine. Based on LC-MS technique, raw data was pre-treated by Lipidsearch software. Lipids were initially identified by retention time and mass-to-charge ratio (m/z). Peak extraction was performed based on predefined thresholds for mass accuracy (ppm), signal-to-noise ratio (S/N), and adduct ions. Peak alignment across different samples was then carried out by correcting for retention time shifts and mass deviations (ppm). Lipids with a coefficient of variation (CV) < 30% in quality control (QC) samples were retained for subsequent quantitative analysis. Differentially expressed lipids were identified based on the following criteria: variable importance in projection (VIP) > 1.0, fold change (FC) > 1.2 or FC < 0.833, and *p*-value < 0.05. Visualization of the differential lipid profiles was performed using volcano plots generated with the R package ggplot2.

### Statistical analysis

3D construction for IF images was proposed by Imaris9.9.0. Western blot and IF were analyzed in the ImageJ. All statistical analyses were performed using GraphPad Prism software (version 9.5.1). Data are presented as mean ± standard deviation (SD) or mean ± standard error of the mean (SEM), as indicated in the figure legends. Comparisons between two groups were analyzed using the unpaired Student’s *t*‑test. For comparisons among three or more groups, one‑way ANOVA was applied. Two-way ANOVA was used for two-factor analyses. Specific statistical methods for each panel are indicated in the corresponding figure legends. Results were considered significant at *p* < 0.05.

## Supplementary Information

Supplementary Material 1.

## Data Availability

The datasets used and analysed during the current study are available from the corresponding author on reasonable request.

## Abbreviations

MS: Multiple sclerosis
BBB: Blood-brain barrier
RRMS: Remitting relapse multiple sclerosis
SPMS: Secondary progressive multiple sclerosis
CNS: Central nervous system
EAE: Experimental autoimmune encephalomyelitis
NETs: Neutrophil extracellular traps
mLVs: Meningeal lymphatic vessels
LEC: Lymphatic endothelial cell
ME: Meningeal enhancement
EDSS: Expanded Disability Status Scale
DMT: Drug modified therapy
Hex1Cer: Hexosylceramide (Monohexoside)
PS: Phosophate serine
Cer: Ceramide
ELISA: Enzyme linked immunosorbent assay
qPCR: Quantitative Real-time polymerase chain reaction
scRNA-seq: Single cell RNA sequencing
LFB: Luxol fast blue
Dpi: Days post immunization
LPC: Lysophosphatidylcholine
