# Supplementary material for: Meningeal lymphatics orchestrate vertebral neutrophils via a Hex1Cer-NETs axis to promote autoimmune neuroinflammation
Source: J Neuroinflammation. 2026 Aug 13;23:365. doi: 10.1186/s12974-026-03994-x (PMC13749699; doi:10.1186/s12974-026-03994-x)
Supplement: Supplementary file 1 [file 12974_2026_3994_MOESM1_ESM.docx]

**Supplementary**

**S1.** (**A**) Flow cytometry gating strategies in the meninges of different stage of EAE. (**B**) Flowcytometry gating strategy of bone marrow cells in granulopoiesis. Granulocyte-macrophage progenitor, metamyelocyte, myelocytes, bands and mature neutrophils during the granulopoiesis were analyzed in EAE mice. Granulopoiesis and migration was significantly up-regulated. (**C**) Flow cytometry analysis revealed that total leukocyte counts in the vertebral bone marrow were significantly elevated in EAE mice but markedly reduced following X-ray irradiation, accompanied by a significant decrease in the proportion of neutrophils. Conversely, in the femoral bone marrow, total leukocyte levels showed no significant change, whereas the percentage of neutrophils after irradiation increased compared with that in healthy controls. Left panels indicate the flow cytometry gating strategy, and right panels show the quantitative statistical analysis. One-way *ANOVA*, N = 7, * *p* < 0.05. (**D**) Flow cytometry gating strategies in the meninges (upper panel) and spinal cord (lower panel) of radiation group. (**E, G**) In spinal cord, after radiation EAE mice showed a lower infiltration as well in total leukocytes, microglia and T cells. One-way *ANOVA*, N = 7, * *p* < 0.05. T cells decreased in the meninges as well. One-way *ANOVA*, N = 7, * *p* < 0.05. (**F**) Representative flow cytometry gating strategy for peripheral blood of X-ray radiation group.

**S2.** (**A**) In the onset stage, CitH3^+^ NETs were mostly occurred around or in the LYVE1^+^ lymphatic vessels. Scale bar = 200μm or 1mm. Blue shading indicates the nerve roots and dorsal root ganglia (DRG), white dashed lines outline the lymphatic vessels and yellow arrows highlight the accumulation of NETs. (**B**) In the spinal cord, MPO^+^CitH3^+^ NETs showed until peak stage after NETs in the meninges in the onset. Scale bar = 20μm. (**C**) Gating strategy of NETs on the meninges. (**D**) NETs increased in the onset and peak of EAE detected by flowcytometry. One-way *ANOVA*, N = 6, * *p* < 0.05, ** *p* < 0.01.

**S3**. (**A**) Feature plot (left) and violin plot (right) suggested higher expression of *S100a8* and *S100a9* after demyelination during the onset and peak stage. Log2FoldChange > 1, *p*.adjust < 0.05. (**B**) However, IF suggested no increase in the protein level of mLECs. Scale bar = 20μm. (**C**) VEGFR3i treatment reduced the infiltration of myeloid cells and macrophages in the meninges as well. One-way *ANOVA*, N = 6, * *p* < 0.05, ** *p* < 0.01. (**D**) T cells didn’t drop significantly in the blood of group VEGFR3i. One-way *ANOVA*, N = 6. **(E)** Gating strategy of Live/Dead⁻CD45⁻CD31⁺LYVE1⁺PDPN⁺ lymphatic endothelial cells in the vertebral meninges. **(F)** Flow cytometry analysis of Live/Dead⁻CD45⁻CD31⁺PDPN⁻ blood vessel endothelial cells (left) and CD45⁺ leukocytes (right) were not influenced by VEGFR3 inhibition efficiency. One-way *ANOVA*, N = 10, ns not significant. **(G)** Gating strategy of immunocytes in EAE of VEGFR3i group.

**S4**. (**A-B**) Drainage of dMBP^+^ myelin debris was analyzed in the LYVE1^+^ meningeal lymphatic vessels and deep cervical lymph nodes (dCLNs) in cuprizone model. After ligation of dCLNs, myelin debris accumulated in the mLVs. Scale bar = 20μm or 100μm. One-way *ANOVA*, N = 3-4, ** *p* < 0.01. (**C**) Western blot showing elevated MPO and CitH3 expression in neutrophils cocultured with myelin debris-pretreated LECs. Experiment was repeated for 3 times. * *p* < 0.05. (**D**) There is up-regulation in oxidative level in mLECs after treated by myelin debris. Scale bar = 100μm. Student’s *t*-test, N = 5, * *p* < 0.05. (**E**) Pathway “fatty acid metabolic process” was enriched in the GSEA. Log2FoldChange > 1, *P.*adjust < 0.05. (**F**) Expression in the pathway “Glycosphingolipid biosynthesis” changed showed by GO enrichment. (**G**) Expression scores for the ceramide synthesis-related gene set in different cells on the meninges. (**H**) Flow cytometry gating strategy for NET release from *in vitro* mouse bone marrow-derived neutrophils (BMDNs). (**I**) Flow cytometry gating strategy in the meninges and spinal cord of the UGT8i series experiment.

**S5**. (**A**) GSK484 (upper) inhibit NETs formation on the meninges through intrathecal injection. Depletion of neutrophils on the meninges by intrathecal injection of anti-Ly6G antibody (bottom). One-way *ANOVA*, N = 6, ** *p* < 0.01. (**B**) Demyelination was attenuated after treatment with GSK484. Scale bar = 100μm. Student’s *t*-test, N = 3, *** *p* < 0.001. (**C**) T cells production dropped in the lymph nodes and blood as well in GSK484 group. One-way *ANOVA*, N = 6, * *p* < 0.05, *** *p* < 0.001. (**D**) Treated by GSK484 reduced total myeloid cells and neutrophils infiltration in the spinal cord but not B cells and dendritic cells. One-way *ANOVA*, N = 6, * *p* < 0.05, ** *p* < 0.01. **(E-F)** Flow cytometry gating strategy in the meninges and spinal cord of the GSK484 and anti-Ly6G series experiment.

**S6.** (**A**) CD19^+^ B cells contacted with LY6G^+^ neutrophils by SELL-SELPLG signal. Scale bar = 10μm. (**B**) Evaluation of the blocking efficiencies of anti-CD62L (left) and anti-CD162 (right) antibodies via a flow cytometry-based competitive binding assay. One-way *ANOVA*, N = 5, * *p* < 0.05, *** *p* < 0.001. (**C**) Quantitative PCR (qPCR) analysis showing downregulated mRNA expressions of *Ccr6* and *Cxcl9* in the spinal cord of EAE mice treated with anti-CD62L antibody, and decreased *Cxcl11* expression in the anti-CD162 group. (*N* = 3 mice per group, with 3 technical replicates per animal). One-way *ANOVA*, N = 6, * *p* < 0.05, ** *p* < 0.01. (**D-E**) Flow cytometry gating strategies in the meninges (D left panel, E) and spinal cord (D right panel) of anti-62L/162 treatment. (**F**) Infiltration of total leukocytes and B cells in meninges and total leukocytes and myeloid cells in the spinal cord was reduced after treatment of anti-CD162 antibody or anti-CD62L antibody. One-way *ANOVA*, N = 6, * *p* < 0.05, ** *p* < 0.01.

**S7**. Flow cytometry gating strategies of sivelestat treatment.

**Supplementary Table 1. Patient demographic data**

| No. | Gender | Age | Meningeal enhancement | EDSS |
| --- | --- | --- | --- | --- |
| 1 | Male | 19 | Yes | 3.5 |
| 2 | Male | 66 | Yes | 1.0 |
| 3 | Female | 40 | Yes | 6.0 |
| 4 | Female | 34 | Yes | 6.0 |
| 5 | Female | 60 | Yes | 2.0 |
| 6 | Female | 16 | Yse | 3.0 |
| 7 | Female | 16 | Yes | 1.5 |
| 8 | Female | 36 | Yes | 2.0 |
| 9 | Female | 25 | Yes | 2.5 |
| 10 | Female | 24 | No | 2.0 |
| 11 | Female | 27 | No | 2.0 |
| 12 | Female | 32 | No | 2.0 |
| 13 | Male | 40 | No | 3.0 |
| 14 | Female | 22 | No | 1.0 |
| 15 | Female | 29 | No | 1.0 |
| 16 | Female | 24 | No | 0.0 |
| 17 | Female | 54 | No | 1.0 |

| Demographic Information | Without meningeal enhancement (*N* =8) | With meningeal enhancement (*N* =9) |
| --- | --- | --- |
| Average age (SD) | 31.5 (10.7) | 34.7 (18.3) |
| Male | 1 | 2 |
| Female | 7 | 7 |
| EDSS in remitting (SD) | 1 (0.8) | 0.6 (1.4) ^ns^ |

*ns No significance.

**Primary western blot image.**

**S6.1** Whole PVDF membrane of Fig 3.E-F from different experiment. The left is the primary image of the middle and the middle was displayed in the figure3E.

Middle,from top to bottom: MPO,GAPDH,CitH3

Right,from top to bottom: MPO,GAPDH,CitH3

**S6.2** Whole PVDF membrane of Fig 4.D. Same membrane. The left was covered while exposed due to the high background intensity.
